# A New and Remarkable Phase in Medical Practice

**Published:** 1891-08

**Authors:** 


### EDITORIAL DEPARTMENT.

F. E. DANIEL, M. D., EDITOR AND PUBLISHER.

THIS JOURNAL, by unanimous vote of the members, was made the Official Organ of the Austin District Medical Society.

COLLABORATORS:

*Dr. R. M. Swearingen, Austin.*
*Prof. B. E. Hadra, M. D., Galveston.*
*Prof. Geo. Cupples, M. D., San Antonio.*
*Dr. T. C. Osborn, Cleburne, Texas.*
*Dr E. J. Doering, Chicago.*
*Dr. E. J. Beall, Fort Worth.*
*Dr Odo Betz, Germany.*
*Prof. W. B. Rogers, M. D., Memphis.*
*Dr. J. W. McLaughlin, Austin.*
*Dr. T. J. Tyner, Austin.*
*Prof. J. F. Y. Paine, M. D., Galveston.*
*Dr. R. H. L. Bibb, Mexico.*
*Dr. T. J. Bennett, Austin.*
*Dr. Bat Smith, Wharton.*
*Dr. E. Meierhof, New York.*
*L. H. Luce, M. D., West Tisbury, Mass.*

### A NEW AND REMARKABLE PHASE IN MEDICAL PRACTICE.

Trust to the genius of the native American to discern the glimmer of the dollar a long way off, and by faith, or by hook or crook to bring it near; to "go for it" in a "way that is peculiar"—ly American.

The latest process of capturing the elusive coin, *i. e.*, as auxiliary to "our noble profession," has been instituted by the Hot Springs, Arkansas, doctors. It is to send out decoys,—*drummers*, who board the incoming train, along with the hotel runners, the baggage check man, the hack man, etc., and capture the pilgrims who journey to that modern Mecca in pursuit of lost health. This they do after the manner of the confidence men and bunco-steerers of New York. They readily diagnose the healthseeker, and steer him into the,—office, (we had nearly said "clutches") of a doctor, who divides with them the fee, (had nearly said "spoils") secured from the captive. (What's the matter with us; we are getting to be irreverent.)

This practice had become so general at Hot Springs, that the city council, who must themselves have some of the aforesaid "genius" for seeing a dollar in the distance, put a tax upon it, —dignified it by calling it an occupation, and now, according to "our esteemed contemporary," the *Journal of the Arkansas Medical Association*, require those fellows who follow it to take out a license and wear a badge! Moreover, these persons are registered, and their names, in connection with those of their employers, are published. Such a list appears in the aforesaid "esteemed contemporary." Some of these doctors have ten, some four or five, and one poor fellow only one drummer. *Did* we see in that list the names of some of the members of the great American Medical Association? or did we not? We were so surprised that, like Bro. Shrady, who, on reading the list, says he rubbed his eyes and wiped the lenses of his glasses and looked again." We rubbed our eyes also, but we are not really certain yet whether there are, or are not, any of the elect engaged in this *new industry*.

But, what if there be? (They are prohibited by the code from advertising, you know.) Are they not actuated by a great zeal in the cause of humanity? Do not our brethren of a kindred profession, to save the souls of the heathen, brave the perils of the sea, and of the African jungles? aye, brave the appetite of those cannibals who, Sydney Smith tells us, always "keep cold missionary on the side-board"? Shall the doctor—the great humanitarian—brave nothing, do nothing to heal the sick? Is this not progress? Is this not coming out of the chrysalis state into the full feather of humanitarianism? Sure, he must find them before he can heal them; and here, then, do we not see an effort to emulate the missionary? Who shall say now that medicine is a trade, or that the ends do not justify the means? Yet, we venture, some cynic will accuse these zealous gentlemen of sordid motives, and outrage their sensibilities by an intimation that they do these things *for money!* Shame! These Hot Springs disciples are doubtless engaged in a great mission of mercy, and vie, the one with the other, in *finding out* cases of "lost manhood," broken down health from early or frequent "indiscretions,"—in speeding to the relief of the modern Lazaruses, (who are gobbled up before they get to the gate, and who, consequently, are no longer to be found waiting at any gate). No doubt they pay these drummers; but as to *their* being paid—(in medicine, you know, the doctor is so modest he bashfully *accepts* an "honorarium")—perish the thought!

But the industry, or philanthropy, more properly, being young yet, and undeveloped, presents many possibilities. Let us suggest one. Let your drummer carry a line of samples of your work, and show what you can do. Photos will do very well, but the "wet specimen," where possible, will be more impressive. Fox's or Piffard's or Morrow's recent illustrated work will be a big thing, and can be made to convey a convincing idea of what the doctor can do in healing the sick. And in the near future a passenger on an ingoing train approaching this station may hear and see something like this:

Steerer (approaching man on crutches)—"Want a doctor, sir? Got any particular preference? No? Well, you are lucky, for I can take you to just the man you want—Dr. Bun Combe, late of London and New York, sir. *Heard* of him, of course,—greatest doctor in the world; makes a specialty of all the ills that flesh is heir to, and cures 'em too; never fails; and don't use no mercury nor potash, neither, like Dr. Bung and Lung and Dung and all them fellows"—(this is the strongest point). And (producing Morrow's Atlas of S. and V. Diseases) "I don't care whether you've got *alopecia areata*, like *that;* or *psoriasis maculata*, like *that;* or *impetigo contagiosa*, like *that:* or *eczema erythematosa*, like *that:* or *syphilo-dermatitis*, like *that;* or just plain old '*ral*, Dr. Bun Combe is your man! and will guarantee a cure, *sure pop!* Step this way, sir, carriage waiting,—no charge; Dr. Bun Combe does the han'some, every time, and don't you forget it." Leads the—patient (had nearly said "victim") off to the—philanthropist (nearly said "robber"), who, like (a spider in his den) Good Samaritan by the roadside, is waiting for his (prey) patient.

We would also suggest that instead of a badge, these auxiliary Good Samaritans be ornamented with a metal collar around the neck, such as their prototypes, the Saxon swine-herders, wore, bearing the name of their master; and that the "doctor" be branded on the forehead, to distinguish the true philanthropist from the sordid herd who actually wait to be sent for.

